# Ergot in Skin Diseases

**Published:** 1883-06

**Authors:** 


### Ergot in Skin Diseases.

Dr. Heitzmann, of New York, had found this agent of service in skin diseases of a congestive character. In some cases of pruritus it had acted like a charm. In certain forms of acne, especially pustular acne of the beard, and in erythema, it is very useful. He use it both internally and locally (the former ʒ ss doses fl. ext.)—*Maryland Med. Jour.*

